# Risk factors of reinfection after prosthesis removal and antibiotic bone cement spacer implantation for the treatment of periprosthetic joint infection

**DOI:** 10.1186/s12879-022-07908-z

**Published:** 2022-12-05

**Authors:** Qingkai Wang, Jincheng Huang, Xiao Chen, Yi Jin

**Affiliations:** 1 Department of Orthopaedics, Henan University People’s Hospital, Henan Provincial People’s Hospital, No. 7, Weiwu Road, Zhengzhou, 450003 Henan Province China; 2 Department of Orthopaedics, Henan Provincial People’s Hospital, Henan University People’s Hospital, Zhengzhou University People’s Hospital, No. 7, Weiwu Road, Henan Province 450003 Zhengzhou, China

**Keywords:** Arthroplasty, Periprosthetic joint infection, Antibiotic bone cement spacer, Two-stage revision, Risk factor

## Abstract

**Background:**

Prosthesis removal and antibiotic bone cement spacer implantation is a very important link in two-stage revision of periprosthetic joint infection (PJI) after artificial joint replacement, which is key to the smooth progress of second-stage revision surgery. There are few reports on the risk factors of reinfection after prosthesis removal and antibiotic bone cement spacer implantation for PJI. This study aimed to investigate the risk factors of reinfection after prosthesis removal and antibiotic bone cement spacer implantation for the treatment of PJI.

**Methods:**

Clinical data of 40 patients who underwent prosthesis removal and antibiotic bone cement spacer implantation for PJI after arthroplasty in our hospital from January 2013 to July 2019 were retrospectively analyzed. During the follow-up period of at least 2 years, 21 patients underwent complete two-stage revision after the removal of the antibiotic bone cement spacer, and 19 patients did not receive a new prosthesis due to other factors, such as reinfection or the patient’s wishes, record the infection control of patients during the treatment. Reinfection after prosthesis removal and antibiotic bone cement spacer implantation was defined as failure of effective control of infection, symptoms of reinfection, requires increased antibiotic therapy or reoperation. Multivariate Cox proportional hazards model was used to analyze the risk factors associated with reinfection after prosthesis removal and antibiotic bone cement spacer implantation.

**Results:**

Of the 40 patients, nine (22.5%) developed reinfection after prosthesis removal and antibiotic bone cement spacer implantation with a mean follow-up duration of 31 months, and multivariate analysis revealed that history of prior revision surgery (hazard ratio [HR] = 6.317, confidence interval [CI]: 1.495–26.700; p = 0.012) and presence of sinus tract before treatment (HR = 5.117, 95% CI: 1.199–21.828; p = 0.027) were independent risk factors for reinfection after prosthesis removal and antibiotic bone cement spacer implantation.

**Conclusion:**

History of prior revision surgery and presence of sinus tract are two independent risk factors for reinfection in patients with PJI treated with prosthesis removal and antibiotic bone cement spacer implantation.

## Background

Periprosthetic joint infection (PJI) is a “catastrophic complication” that could occur after artificial joint replacement, and could impose a heavy burden on patients and the society [1, 2]. Reports have shown that the incidence of periprosthetic hip joint infection is 1% [3], while the incidence of periprosthetic knee joint infection is 1–2% [4]. PJI has been reported to be the most common cause of failure in total knee arthroplasty (TKA), and is also the third most common cause of failure after total hip arthroplasty (THA) [5]. With the development of artificial joint replacement technology and the increasing age of the population, it is believed that an increasing number of patients with surgical indications will choose this technique for the management of advanced joint diseases, and PJI will become more widespread. Therefore, more attention should be paid to the treatment of PJI. The main treatment modalities of PJI include debridement, antibiotics and implant retention (DAIR), one-stage revision, two-stage revision, arthrodesis and amputation [6]. Among them, most scholars believe that two-stage revision arthroplasty using an antibiotic bone cement spacer has become the gold standard for the management of chronic PJI [7].

Currently, the active centralizer of antibiotic bone cement spacer used in clinical practice can not only release high concentrations of antibiotics locally, but also reduce scar formation, stabilize soft tissue tension, avoid further bone loss, and improve joint function and the quality of life of patients during the spacer period [8, 9]. Moreover, if the patient’s pain is relieved and joint function significantly improves, some patients may consider retaining the antibiotic bone cement spacer due to their own conditions rather than implanting new prostheses. However, even with continuous use of systemic and local antibiotics, the non- bioactive nature of the cement spacer remains a potential interface for pathogens to form biofilms and cause recurrent infections [10]. It is also unclear whether pathogens remain on the surface of the cement spacer and in local soft tissues of joints when patients undergo second-stage revision surgery.

The criteria for successful PJI treatment should be the eradication of the infection and restoration of joint function. However, the effect of PJI treatment is not satisfactory, and many patients have recurrent infection during or after treatment, therefore, it is of interest to investigate the risk factors related to treatment failure in the treatment of PJI. Prosthesis removal and antibiotic bone cement spacer implantation is key to the smooth progress of two-stage revision surgery for PJI after artificial joint replacement. Several studies have investigated the risk factors of reinfection after DAIR or two-stage revision. However, few scholars have studied the situation for patients after first-stage prosthesis removal and antibiotic bone cement spacer implantation in two-stage revision surgery.The purpose of this study was to investigate the risk factors for reinfection after prosthesis removal and antibiotic bone cement spacer implantation for the treatment of PJI and hoping to provide help for the smooth progress of two-stage revision arthroplasty.

## Methods

### Inclusion and exclusion criteria

#### Inclusion criteria

The inclusion criteria included the following: (i) after total hip and knee arthroplasty, we identified patients with PJI who met the 2011 American Muscular Skeletal Infection Society (MSIS) diagnostic criteria; (ii) patients who received prosthesis removal and antibiotic bone cement spacer implantation in our hospital due to PJI; (iii) patients with reinfection that was diagnosed by our hospital or those who met the 2011 edition of the MSIS diagnostic criteria; and (iv) patients with complete follow-up data.

#### Exclusion criteria

The exclusion criteria included the following: (i) cases in which the follow-up time duration was less than 2 years; (ii) patients with incomplete case data; (iii) patients with unconfirmed reinfection; (iv) cases in which the follow-up was interrupted or the patient was uncooperative; (v) the use of DAIR; (vi) patients who underwent revision surgery before reinfection after prosthesis removal and antibiotic bone cement spacer implantation ; (vii) cases in which the PJI diagnosis was clear and no surgery was performed; (viii) artificial joint replacement due to bone tumor disease; and (ix) cases in which microbiological culture results were unreliable.

### General information of the participants

**Fig. 1:**
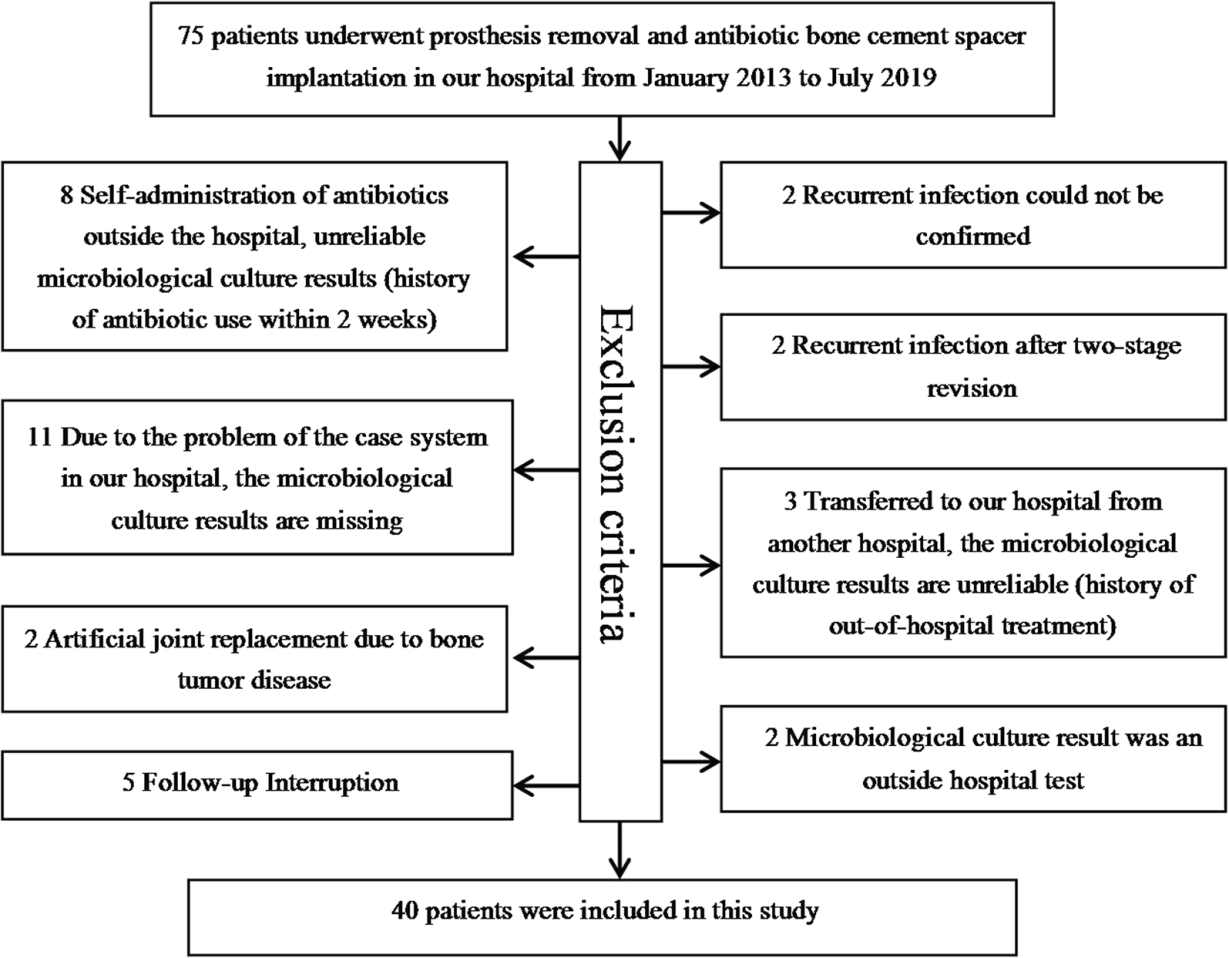
Flow diagram of included patients

After Institutional Review Board approval, we retrospectively reviewed patients who underwent prosthesis removal and antibiotic bone cement spacer implantation for PJI after arthroplasty in our hospital from January 2013 to July 2019. A total of 40 patients were included in this study according to the inclusion and exclusion criteria (Fig. 1). Among them, 19 patients underwent THA and 21 patients underwent TKA. There were 18 male and 22 female participants, aged (65.85 ± 12.37) years (range: 40–86 years) (Table 1).

**Table 1:** Comparison of general data of patients with recurrent infection after prosthesis removal and antibiotic bone cement spacer implantation

	Risk factors	No reinfection group (N = 31)	Reinfection group (N = 9)	p value
Patient characteristics	Age (years)			
	≥ 65	23 (74%)	6 (67%)	–
	< 65	8 (26%)	3 (33%)	0.6861
	Female	17 (54%)	5 (56%)	0.9999
	Smoking history	5 (16%)	0 (0%)	0.5696
	Alcohol history	6 (19%)	0 (0%)	0.3065
	ASA grade			
	1–2	17 (55%)	6 (67%)	–
	3–4	13 (42%)	3 (33%)	0.7110
	aCCI score			
	< 3	19 (61%)	4 (44%)	–
	≥ 3	12 (39%)	5 (56%)	0.4561
Medical conditions	Diabetes	5 (16%)	2 (22%)	0.6446
	Hypertension	10 (32%)	1 (11%)	0.3994
	Coronary heart disease	4 (13%)	1 (11%)	0.9999
	Rheumatoid arthritis	1 (3%)	0 (0%)	0.9999
Microbiology	Culture negative	12 (39%)	4 (44%)	0.9999
	Gram-positive bacteria	10 (32%)	4 (44%)	0.6935
	Gram-negative bacteria	4 (13%)	1 (11%)	0.9999
	Fungi	1 (3%)	0 (0%)	0.9999
	Co-infection	4 (13%)	0 (0%)	0.5573
Other risk factors	Duration of infection			
	< 3 Months	9 (29%)	6 (67%)	–
	≥ 3 Months	22 (71%)	3 (33%)	0.0572
	History of prior Infection	2 (6%)	1 (11%)	0.5450
	History of prior revision surgery	1 (3%)	4 (44%)	0.0061
	Presence of sinus tract	8 (26%)	6 (67%)	0.0444

ASA grade: American Society of Anesthesiologists Physical Status Classification System, aCCI score: Age-adjusted Charlson Comorbidity Index, Gram-positive bacteria: Staphylococcus aureus, Staphylococcus haemolyticus, Staphylococcus epidermidis; Gram-negative bacteria: Escherichia coli, Klebsiella pneumonia, Pseudomonas aeruginosa, Enterobacter cloacae; Fungi: Candida parapsilosis; Co-infection: Pseudomonas putida andMobility baumannii, Staphylococcus aureus and Mobilus baumannii and Calcoaceticus complex, Pseudomonas aeruginosa and Staphylococcus epidermidis, Staphylococcus epidermidis and Rhizobium radiobacter

### Diagnostic criteria for periprosthetic Joint infection

According to the diagnostic criteria of MSIS (2011 Edition), the diagnosis of PJI should meet one of the following three conditions: (1) there is a sinus tract communicating with the prosthesis on the surface of the skin over the joint; (2) the same pathogen is obtained twice after culturing synovial fluid or tissue samples that were collected independently from the diseased joint; (3) four of the following six criteria are met: (a) elevated serum erythrocyte sedimentation rate (ESR) and serum C-reactive protein (CRP) level; (b) increased synovial fluid white blood cell count; (c) increased the percentage of multinucleated cells in synovial fluid; (d) presence of pus in the joint; (e) the culture result of pathogenic microorganisms in the tissue or synovial fluid sample is positive; and (f) the average number of neutrophils in five high-power (× 400) fields was found to be greater than five during the pathological analysis of periprosthetic tissue [11].

### Study method

All patients with PJI in this study were diagnosed according to the diagnostic criteria of our hospital, and joint prosthesis removal and antibiotic bone cement spacer implantation were performed by the Department of Orthopedics of our hospital. Variables that may affect the treatment outcome of PJI were then collected according to previous literature studies and combined with the completeness of institutional data, including patient characteristics, medical diseases, microbiological examination after admission, and other possible risk factors, then univariate analysis was performed for the risk factors of recurrent infection after treatment of PJI had been confirmed [12–16]. Patients were divided into the reinfection group and the non-reinfection group according to whether they had reinfection after prosthesis removal and antibiotic bone cement spacer implantation. The no-reinfection group was defined as having been followed-up for at least 2 years without any evidence of reinfection found by the patients, the reinfection group was defined as cases in which an initial infection that was not effectively controlled, or mixed infection formed by new pathogens occurred, all of which eventually developed symptoms of reinfection and require additional antibiotic therapy or reoperation. In this group of patients, the presence of reinfection was the end point of our observation. It should be mentioned that we studied the risk factors of reinfection after prosthesis removal and antibiotic bone cement spacer implantation in patients with PJI, and the ultimate aim of these patients was to receive a new prosthesis after the infection was controlled. The time of antibiotic bone cement spacer placement was determined by when the infection was controlled; therefore some patients in the group without recurrence of infection underwent a second stage of revision surgery during the follow-up period; the remaining patients chose to retain the spacer according to their personal wishes and were not implanted with a new prosthesis.In conclusion, none of the patients in this group experienced reinfection during a minimum follow-up duration of 2 years. None of the patients in the reinfection group underwent a second stage of revision surgery before the end of the observation period to ensure that all patients in this group only underwent prosthesis removal and antibiotic bone cement spacer implantation.

### Surgical process

**Fig. 2:**
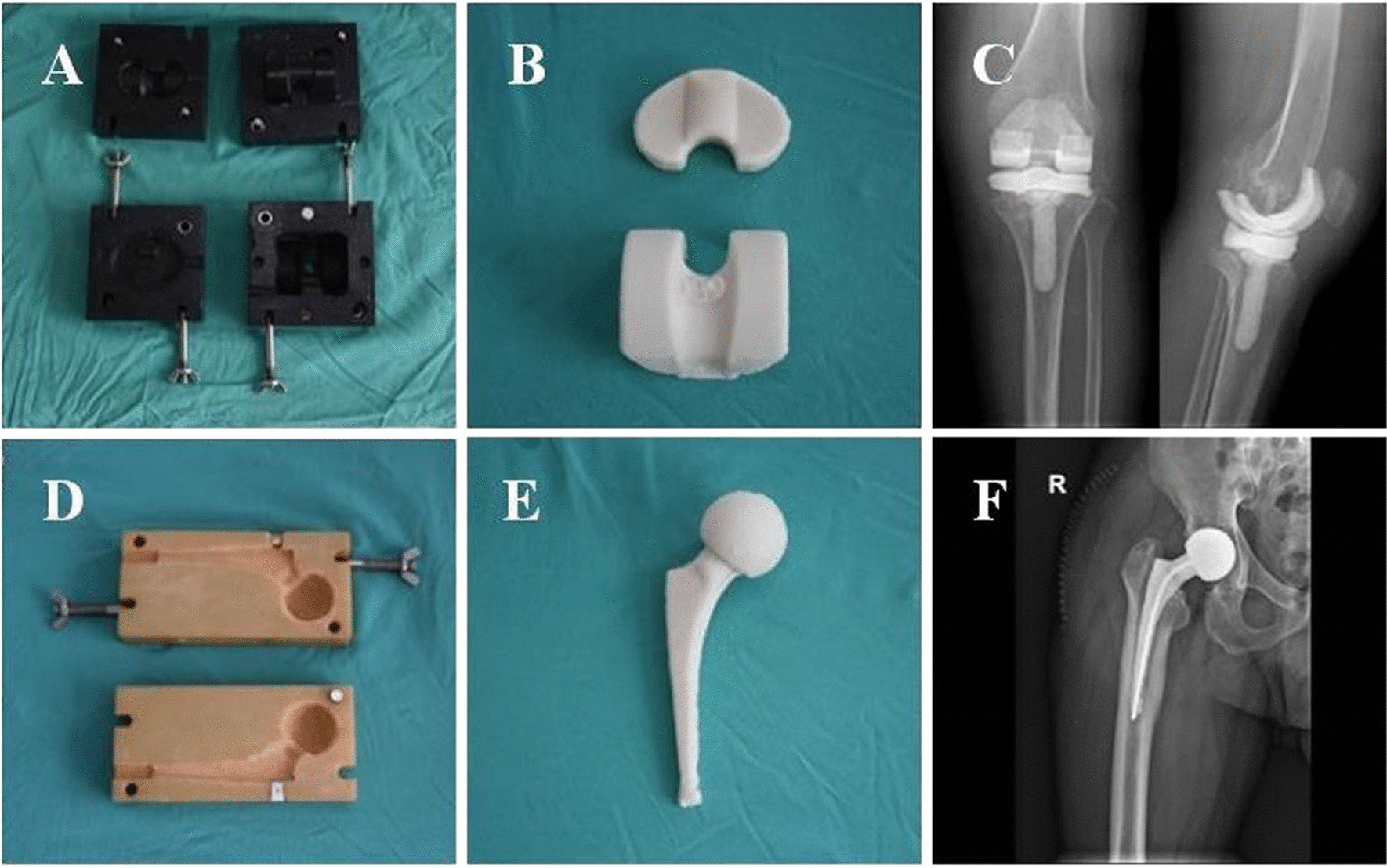
Fabrication of joint type antibiotic bone cement spacer. Self-made knee joint spacer mold imitating joint shape (**A**). Joint type knee antibiotic cement spacer made from mold (**B**). Anteroposterior and lateral radiographs of the left knee joint after spacer implantation (**C**). Self-made hip joint spacer mold imitating joint shape (**D**). Joint type hip antibiotic cement spacer made from mold (**E**). Anteroposterior radiograph of the right hip joint after spacer implantation (**F**)

Stage I: Excision of sinus tracts, safe removal of the original prosthesis, thorough cleaning of periprosthetic inflammatory necrotic tissue and secretions, intraoperative removal of at least three tissue samples at different sites for rapid frozen pathological examination and bacterial culture, and then implantation of antibiotic bone cement centraliser.Antibiotic regimens in bone cement were determined based on preoperative etiologic culture results. If the preoperative etiological culture was gram-positive, 6 g vancomycin and 2 g imipenem and cilastatin sodium per 80 g package of bone cement was used; however, if the preoperative etiological culture was gram-negative, 2 g vancomycin and 6 g imipenem and cilastatin sodium per 80 g package of bone cement was used. If the preoperative etiological culture was negative or if there was a co-infection, 6 g vancomycin and 2 g imipenem and cilastatin sodium per 80 g package of bone cement was used, while if the preoperative etiological culture was fungal, 8 g amphotericin per 80 g package of bone cement was used. Antibiotics to which the cultured organisms were susceptible were administered intravenously for 1–2 weeks postoperatively (vancomycin was used if culture was negative) based on susceptibility test results, followed by orally sensitive antibiotics for 4–6 weeks (ciprofloxacin and rifampicin were administered orally if culture was negative). Stage II: At least 3 months after prosthesis removal and antibiotic bone cement spacer implantation, we determined whether the patient’s infection was controlled by observing the patient’s clinical symptoms and serological parameters (blood routine, CRP and ESR). Infection disappeared and serological indicators decreased to normal, if serological indicators could not be reduced to normal due to underlying disease, the managing team determined whether the patient could receive two-stage revision surgery based on the patient’s clinical symptoms and the downward trend of serological indicators after discontinuing oral antibiotics. A new prosthesis was then implanted according to the patient’s wishes, as we found that some patients felt that the mobile cement spacer was well used and did not need to undergo a second-stage revision surgery again. Details of the antibiotic bone cement spacers used are shown in Fig. 2

### Statistical analysis

Statistical analysis was performed using SPSS statistical software version 21.0 (Armonk, NY: IBM Corp. USA).The measurement data of the normal distribution are expressed as x ± s, and the measurement data of the skewed distribution are expressed as M (range). Enumeration data were expressed as frequencies or percentages, and Fisher’s exact test was used for comparisons. The Kaplan-Meier method was used to calculate the survival rate and draw the survival curve. The log-rank test was used for the univariate analysis. P < 0.05 was considered statistically significant.Variables with statistically significant differences were included in the Cox model for multivariate analysis.

## Results

All patients received complete follow-up, with an average follow-up time of 31.33 ± 16.54 months (range: 0.49–52.50 months). Of the 40 patients, nine had reinfections after prosthesis removal and antibiotic bone cement spacer implantation, and 31 had no reinfection after at least 2 years of follow-up. Of the patients in the group without reinfection, 21 completed the second stage of revision after the infection was controlled and 10 did not undergo the second stage of surgical treatment, the mean interim period for patients undergoing complete two-stage revision surgery was 5.47 ± 3.44 months (range: 3.02–14.42 months). Among patients in the group with reinfection, five underwent prosthesis removal and antibiotic bone cement spacer implantation again, one underwent debridement, one died, and two did not undergo further surgical treatment. Our study included patients who underwent uneventful two-stage revision and all had satisfactory outcomes during follow-up, as we excluded those who had reinfection after two-stage revision because we were unsure of whether the reinfection in these patients was the result of a two-stage revision or an unsuccessful prosthesis removal and antibiotic bone cement spacer implantation at the first stage.

The general data comparison between the reinfection and no reinfection groups is shown in Table 1. Table 2 shows the results of univariate analysis, including the duration of infection (χ^2^ = 5.299, p = 0.0213), history of prior revision surgery (χ^2^ = 15.361, p = 0.0001), and presence of sinus tract (χ^2^ = 4.575, p = 0.0324). Multivariate analysis including statistically significant risk factors in the Cox model revealed that a history of prior revision surgery (hazard ratio [HR] = 6.317, 95% confidence interval [CI]:1.495–26.700; p = 0.012) and the presence of sinus tract (HR = 5.117, 95% CI: 1.199–21.828; p = 0.027) were independent risk factors (Table 3).

**Table 2:** Single factor analysis of recurrent infection after prosthesis removal and antibiotic bone cement spacer implantation

	Risk factors	No reinfection group (N = 31)	Reinfection group (N = 9)	χ^2^	p value
Patient characteristics	Age (years)				
	≥ 65	23 (74%)	6 (67%)	–	–
	< 65	8 (26%)	3 (33%)	0.094	0.7587
	aCCI score				
	< 3	19 (61%)	4 (44%)	–	–
	≥ 3	12 (39%)	5 (56%)	0.758	0.3839
Medical conditions	Diabetes	5 (16%)	2 (22%)	0.227	0.6335
Microbiology	Culture negative	12 (39%)	4 (44%)	0.051	0.8217
	Gram-negative bacteria	4 (13%)	1 (11%)	0.001	0.9748
Other risk factors	Duration of infection				
	< 3 Months	9 (29%)	6 (67%)	–	–
	≥ 3 Months	22 (71%)	3 (33%)	5.299	0.0213
	History of prior Infection	2 (6%)	1 (11%)	0.227	0.6337
	History of prior revision surgery	1 (3%)	4 (44%)	15.361	0.0001
	Presence of sinus tract	8 (26%)	6 (67%)	4.575	0.0324

aCCI score: Age-adjusted Charlson Comorbidity Index; Gram-negative bacteria: Escherichia coli, Klebsiella pneumonia, Pseudomonas aeruginosa, Enterobacter cloacae

**Table 3:** Multivariate analysis of recurrent infection after prosthesis removal and antibiotic bone cement spacer implantation

Risk factors	b	SE (b)	Wald χ^2^	HR	95%CI	p value
Infection duration < 3 months	1.333	0.756	3.111	3.792	0.862–16.674	0.078
History of prior revision surgery	1.843	0.735	6.282	6.317	1.495–26.700	0.012
Presence of sinus tract	1.633	0.740	4.865	5.117	1.199–21.828	0.027

**Fig. 3:**
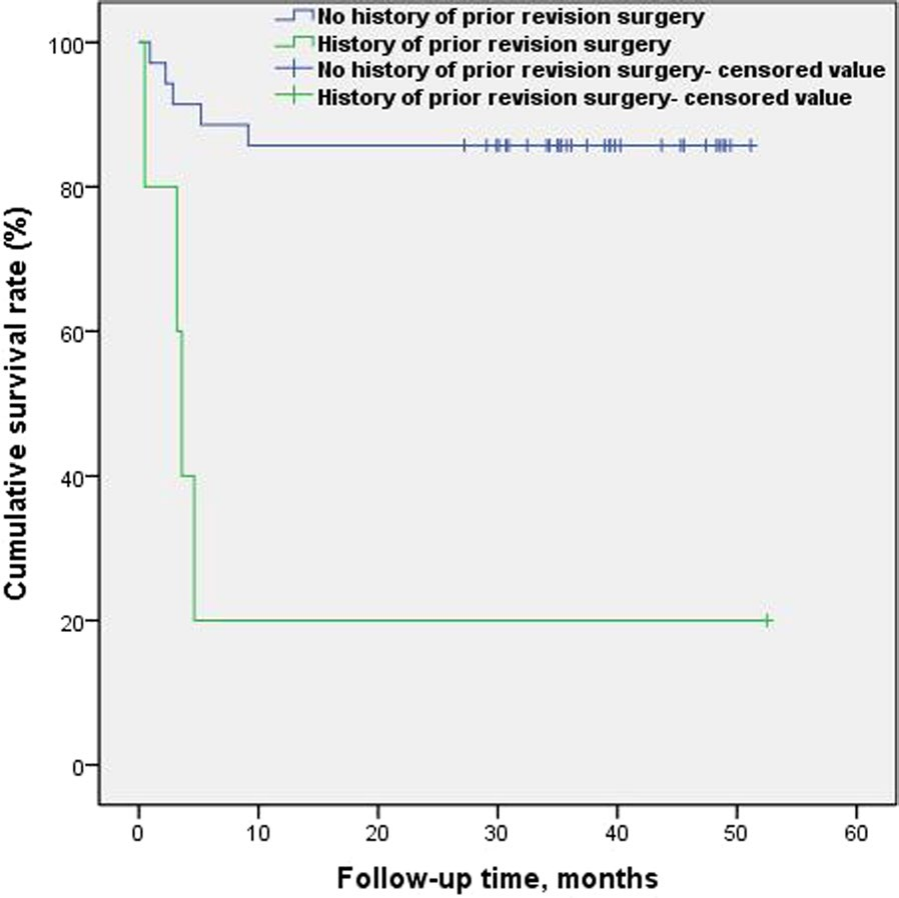
Log-rank test for Kaplan-Meier survival analysis in history of prior revision surgery (p = 0.0001)

**Fig. 4:**
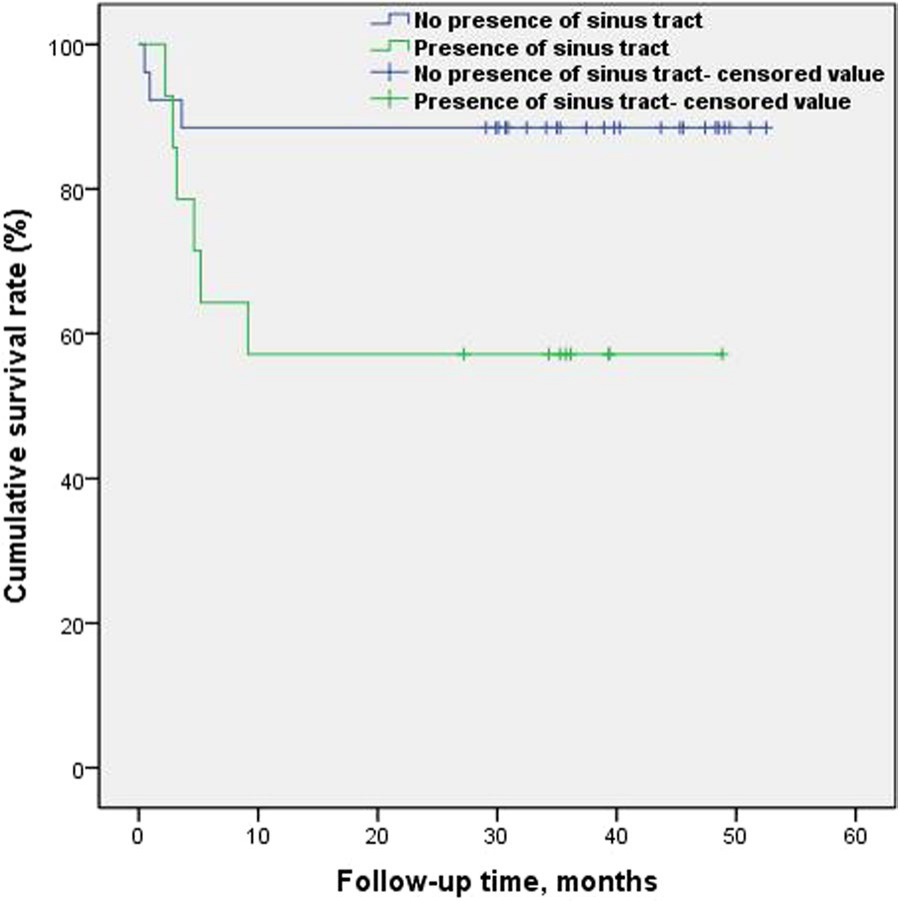
Log-rank test for Kaplan-Meier survival analysis in presence of sinus tract (p = 0.0324)

The time to reinfection after primary prosthesis removal and antibiotic bone cement spacer implantation for the treatment of periprosthetic joint replacement infection were as follows: in 2 cases, recurrent infections occurred 1 month after surgery, in 7 cases recurrent infections occurred 5 months after surgery, and in 9 cases recurrent infections were observed 10 months after surgery. The survival rates of patients with no recurrent infection 1, 5, and 10 months after prosthesis removal and antibiotic bone cement spacer implantation were 95.0%, 82.5%, and 77.5%, respectively, and the survival rates calculated using the Kaplan-Meier method and plotted using log-rank test showed that patients with PJI who had the following risk factors, history of prior revision surgery (80%, 20%, 20%; p = 0.0001, Fig. 3) and presence of sinus tract (100%, 71.4%, 57.1%; p = 0.0324, Fig. 4), at 1, 5, and 10 months after prosthesis removal and antibiotic bone cement spacer implantation, had significantly reduced survival rates for no recurrent infection. The survival rates without reinfection of patients who did not have a history of prior revision surgery at 1, 5, and 10 months after prosthesis removal and antibiotic bone cement spacer implantation were 97.1%, 91.4%, and 85.7%, respectively.The survival rates without reinfection of patients who had a history of prior revision surgery at 1, 5, and 10 months after prosthesis removal and antibiotic bone cement spacer implantation were 80%, 20%, and 20%, respectively.The survival rates without reinfection of patients who without presence of sinus tract at 1, 5, and 10 months after prosthesis removal and antibiotic bone cement spacer implantation were 92.3%, 88.5%, and 88.5%, respectively.The survival rates without reinfection of patients who with presence of sinus tract at 1, 5, and 10 months after prosthesis removal and antibiotic bone cement spacer implantation were 100%, 71.4%, and 57.1%, respectively.

## Discussion

With the development of artificial joint technology, two-stage revision using antibiotic cement spacer is considered the gold standard for the treatment of chronic PJI and has now become an option for many orthopedic surgeons [7]. Although the implanted temporary spacers of antibiotic bone cement can continuously release effective concentrations of antibiotics at the site of infection,we found that some patients with periprosthetic infection still had reinfection after prosthesis removal and antibiotic bone cement spacer implantation. It has also been shown that Staphylococcus species isolates that can grow and have the ability to form biofilms are found on cement beads containing antibiotics [17]. This phenomenon has attracted our attention, and as a very important link in two-stage revision arthroplasty, the success of one-stage prosthesis removal and antibiotic bone cement spacer implantation is key to the smooth progress of the second-stage revision surgery; it is of great clinical significance to investigate the risk factors for recurrent infection after prosthesis removal and antibiotic bone cement spacer implantation, and reasonable selection of antibiotics according to existing risk factors, as this may help to improve the success rate of two-stage revision surgery. The results of this study showed that prior history of revision surgery (p = 0.012) and presence of sinus tract (p = 0.027) were independent risk factors for recurrent infection after prosthesis removal and antibiotic bone cement spacer implantation for PJI, while patients with PJI who had an infection duration of less than 3 months were only significant in the univariate analysis.

There are few reports on the risk factors for reinfection after prosthesis removal and antibiotic bone cement spacer implantation, and most articles only briefly describe the treatment method for patients with reinfection. In a study of 82 patients with PJI after THA who underwent two-stage revision, Toulson et al. found that eight patients developed reinfection before reimplantation of the prosthesis, with a first-stage success rate of 90% [18]. Fink et al. reported in a prospective study of 36 patients that included one patient who required a second prosthesis removal and antibiotic bone cement spacer implantation to control infection before undergoing a second-stage revision, also with a first-stage success rate of over 90% [19]. Lee et al. retrospectively found that two out of 19 patients underwent additional debridement before prosthesis reimplantation due to symptoms of reinfection after prosthesis removal and antibiotic bone cement spacer implantation, with a first-stage success rate of 89.5% [20]. In our study, 40 patients with PJI were retrospectively analyzed, and in nine cases the procedures failed after one-stage prosthesis removal and antibiotic bone cement spacer implantation, with a final success rate of 77.5%. The reinfection rate was significantly higher than that in other scholars’ studies. This may be related to the following factors: First, the cases retrospectively collected in this study were all patients who underwent prosthesis removal and antibiotic bone cement spacer implantation, and the criterion of undergoing a complete two-stage revision was not considered as the main criterion. Second, we believe that this may also be related to the small number of cases included. Therefore, studies with larger sample sizes are needed to further investigate the therapeutic effect of one-stage implantation of antibiotic cement spacer for periprosthetic infection and its impact on two-stage revision surgery.

The results of this study showed that patients with history of prior revision surgery had an increased risk of failure after the first stage of prosthesis removal and antibiotic bone cement spacer implantation. This may be because revision surgery scars local tissue and affects local blood supply; hence the body’s immune response is frustrated, and the anti-infective ability of this system is reduced. Some scholars also believe that many aseptic revisions are actually periprosthetic infections that are not definitively diagnosed by testing [21], this is in line with the findings of Bongartz et al., who showed that patients with a history of periprosthetic infection are at risk of reinfection after treatment [22]. Petis et al. followed 179 patients with chronic PJI who underwent two-stage revision surgery and found that previous revision surgery (HR, 2.8; 95% CI, 1.5–5.2; p < 0.01) was a risk factor for reinfection after risk factor analysis [23]. Similar results were obtained in another study, which found that previous history of revision surgery(adjusted OR, 2.55; 95% CI, 1.22–5.32; p = 0.013) also played a negative role in DAIR for PJI [12]. Jämsen et al. retrospectively reviewed 387 patients who underwent reoperation for PJI and found that both partial and total revision TKA were associated with an increased risk of infection compared to the risk after primary TKA [24].Some scholars also believe that if there is a history of prior revision surgery in the joint undergoing arthroplasty, the risk of postoperative infection will be significantly increased [22].

Some patients with periprosthetic infection will have typical clinical manifestations of the sinus tract, and it has been shown that nearly one-third of patients with periprosthetic infection will develop sinus tracts [25, 26]. The sinus tract, as the main standard, plays an important role in the diagnosis of periprosthetic infection, while the therapeutic effect of the sinus tract on periprosthetic infection is also worthy of attention. A recent study demonstrated that the presence of sinus tracts adversely affect treatment outcomes in patients with periprosthetic infections [13]. Marculescu et al. retrospectively analyzed 91 patients with PJI who underwent DAIR, and multivariate analysis showed that the presence of sinus tract (HR 2.84; 95% CI 1.48–5.44; p = 0.002) was an independent risk factors for treatment failure [27]. Fu et al. retrospectively analyzed 81 patients with PJI after knee arthroplasty who underwent two-stage revision, their study showed that the presence of sinus tract before surgery was also a risk factor for reinfection after two-stage revision [28]. In our study, eight patients had sinus tracts in the non-reinfection group, while six patients had sinus tracts in the reinfection group. After risk factor analysis, we found that the presence of sinus tract was an independent risk factor for prosthesis removal and antibiotic bone cement spacer implantation failure (HR = 5.117, 95% CI: 1.199 − 21.828; p = 0.027). The specific reasons the presence of sinus tracts affects the treatment outcome of periprosthetic infections are currently unknown. Xu et al. proposed some possible explanations, and they concluded that patients with sinus tracts tend to have poor soft tissue conditions and may require additional procedures during surgery [13]. Therefore, we believe that sinus tract formation has a non-negligible influence on the treatment of periprosthetic infection, and surgeons should be aware of this effect.

This study also has some limitations.First, this was a single-center retrospective study with a small number of included cases and possible selection bias.Second, this study included periprosthetic hip infection and periprosthetic knee infection, and readers should be aware of the difference between the two joints.Third, the follow-up duration was short, and if the follow-up duration is increased, the reinfection rate may increase.Fourth, this study had a large time span, which could not remove the difference in results caused by different surgeons.

## Conclusion

In conclusion, we found that PJI patients with a history of prior revision surgery and the presence of sinus tract were significantly related to the risks of reinfection after prosthesis removal and antibiotic bone cement spacer implantation. Effectively reducing the incidence of reinfection in such patients may improve the success rate of two-stage revision arthroplasty and patients’ satisfaction postoperatively.

## Data Availability

The datasets used and/or analysed during the current study are available from the corresponding author on reasonable request.

## Abbreviations

PJI: Periprosthetic joint infection
TKA: Total knee arthroplasty
THA: Total hip arthroplasty
DAIR: Debridement, antibiotics and implant retention
MSIS: Muscular Skeletal Infection Society
ESR: Erythrocyte sedimentation rate
CRP: C-reactive protein
